# Effects of Anticancer Therapy on Osteoporosis in Breast Cancer Patients: A Nationwide Study Using Data from the National Health Insurance Service-National Health Information Database

**DOI:** 10.3390/jcm14030732

**Published:** 2025-01-23

**Authors:** Minji Kwon, Bo-Hyung Kim, Sun Young Min, Sumin Chae

**Affiliations:** 1 Department of Clinical Pharmacology and Therapeutics, Kyung Hee University Hospital, Seoul 02447, Republic of Korea; mkwon1201@gmail.com (M.K.); bhkim98@gmail.com (B.-H.K.); 2 East-West Medical Research Institute, Kyung Hee University, Seoul 02447, Republic of Korea; 3 Department of Surgery, Kyung Hee University College of Medicine, Kyung Hee University Hospital, Seoul 02447, Republic of Korea; breastdrmin@gmail.com

**Keywords:** breast cancer, anticancer therapy, estrogen, osteoporosis

## Abstract

**Background/Objectives**: This nationwide retrospective study evaluated the effects of anticancer therapy on osteoporosis in 126,132 Korean breast cancer survivors from 2002 to 2020. **Methods**: The Cox proportional hazards model assessed the effects of treatment on osteoporosis. To circumvent the guarantee-time bias for osteoporosis development, a landmark analysis was employed. A stabilized inverse probability of treatment weighting was performed to control any confounding bias. The propensity score was calculated using a multinomial logistic regression model with age, national health insurance, and the Charlson comorbidity index. **Results**: During a median follow-up of 4.22 years, 28,603 cases of osteoporosis were documented. Aromatase inhibitors (AIs) were associated with a higher risk of osteoporosis development in comparison to tamoxifen (TMX) or chemotherapy. Notably, AIs administered subsequent to a combination of chemotherapy and anti-HER2 therapy exhibited the highest risk of osteoporosis development. Subgroup analysis revealed that the mean interval from breast cancer diagnosis to osteoporosis development was 5.00 years for women diagnosed with cancer at age < 50 and 3.89 years for those diagnosed at age ≥ 60. TMX increased the risk of osteoporosis in women diagnosed with cancer at age < 50, whereas chemotherapy was not a significant risk factor for osteoporosis development in those diagnosed at age ≥ 60. The impact of anticancer therapy on osteoporosis development was more pronounced in women diagnosed with breast cancer at a younger age compared to those diagnosed at an older age. **Conclusions**: Effective prevention and active management strategies should be implemented to address bone loss in both younger and older breast cancer patients.

## 1. Introduction

Breast cancer is the most common cancer among women globally, with significant advancements in treatment options leading to improved survival rates. The 5-year relative survival rate for Korean patients with breast cancer from 2015 to 2019 was 93.6% [1]. However, breast cancer treatments, particularly hormonal therapy such as aromatase inhibitors (AIs) and chemotherapy, are associated with adverse effects. Among the various adverse effects associated with breast cancer treatment, osteoporosis represents one of the most prevalent and long-term adverse consequences [2]. The risk of bone loss is approximately 70% higher in breast cancer survivors than in cancer-free women. Furthermore, it is estimated that approximately half of postmenopausal breast cancer survivors aged ≥50 years are anticipated to experience an osteoporosis-related fracture during their lifetimes [3,4,5,6].

Osteoporosis is a disease that affects bone mass and microarchitecture, resulting in poor quality of life in postmenopausal women. Estrogen plays a significant role in regulating bone metabolism. As a consequence of the age-related decline in estrogen levels, women are predisposed to developing primary osteoporosis 10 to 15 years after menopause. On the other hand, breast cancer treatments have been demonstrated to cause hypogonadism and accelerate osteoporosis within a relatively short period of time, even in younger women [7,8]. Osteoporosis can have a number of physical and psychological consequences, including body deformity, reduced mobility, fear of future fractures, and depression due to loss of independence [9]. Many patients with osteoporotic fractures require assistance with activities of daily living, which can put a strain on the healthcare system and their families [10]. In addition, hospitalization, surgery, and long-term rehabilitation, especially after femoral and spine fractures, result in significant healthcare costs [11,12]. Furthermore, the economic burden of osteoporosis is not limited to the individual, as it can also impose a financial strain on society through the loss of productivity and early retirement of patients and caregivers [13]. Consequently, the development of effective public health strategies to prevent, early detect, and manage osteoporosis is imperative.

The selection of medical treatments for breast cancers is based on the biological subtypes of the cancer, as determined by hormone receptors and human epidermal growth factor receptor 2 (HER2) status. Cancers that do not express estrogen receptors (ERs) or progesterone receptors (PRs) are classified as “hormone receptor-negative” and require chemotherapy unless the cancer is very small. The chemotherapy regimens available today are highly effective in reducing the chances of cancer spread or recurrence. Chemotherapy involves the use of drugs to destroy or slow the growth of rapidly dividing cancer cells, and these drugs can be administered intravenously or orally, depending on the drug and treatment plan. Common side effects of chemotherapy for breast cancer include fatigue, nausea, vomiting, hair loss, and an increased risk of infection due to lowered white blood cell counts. Long-term side effects can include osteoporosis [14]. Chemotherapy drugs, which are frequently employed in the treatment of breast cancer, induce osteoporosis by damaging the ovaries through a variety of mechanisms. Cyclophosphamide induces DNA double-strand breaks, platinum-based compounds bind to DNA and form cross-links, anthracycline antibiotics inhibit DNA synthesis and function, and adriamycin can cause interstitial fibrosis and ovarian vascular abnormalities in a dose-dependent manner [15]. Of these, cyclophosphamide has the highest risk of chemotherapy-induced ovarian failure (CIOF), followed by platinums, anthracyclines, and taxanes [16].

Breast cancers that express ERs and/or PRs are more likely to respond to endocrine therapy, including tamoxifen and aromatase inhibitors (AIs). Adjuvant endocrine therapy constitutes a pivotal treatment strategy to reduce the risk of recurrence and mortality in women diagnosed with ER-positive disease. These medications are administered orally for a period of five to ten years, reducing the likelihood of these cancers recurring by approximately 50% [17].

AIs, which block the rate-limiting step in estrogen biosynthesis, constitute the standard of treatment for postmenopausal breast cancer patients. AIs markedly reduce estrogen levels and stimulate bone turnover and osteoclast activity, thereby increasing the risk of bone loss and fracture [3,18]. Tamoxifen (TMX), a selective estrogen receptor modulator (SERM), blocks ERs on breast cancer cells and is used in both premenopausal and postmenopausal breast cancer patients. Common side effects of TMX include hot flashes, night sweats, vaginal dryness, and an increased risk of blood clots. However, TMX may also exert a protective effect on bone health, though this is dependent on menopausal status [19,20,21].

Breast cancers that overexpress HER2 protein are amenable to treatment with targeted biological agents such as trastuzumab. The HER2 protein has been demonstrated to promote the growth of cancer cells; consequently, therapies have been developed to target this protein. Trastuzumab, a monoclonal antibody that binds to the HER2 protein expressed on cancer cells, has been shown to inhibit cell growth and enhance immune system-mediated destruction of these cells [22,23]. The efficacy of targeted biological therapies is enhanced when administered in combination with chemotherapy, thereby ensuring the effective eradication of cancer cells. The potential adverse effects of these therapies include cardiovascular complications, infusion reactions, and diarrhea [24]. However, the impact on osteoporosis remains to be elucidated.

Several guidelines recommend the assessment of bone mineral density (BMD) at baseline and periodic intervals in women with breast cancer receiving AIs or experiencing ovarian failure who are at risk of osteoporosis [3,4,6,25,26]. It is challenging to ascertain the association between osteoporosis and breast cancer treatment due to the variability in the impact of treatment on osteoporosis and the complexity of treatment for individual breast cancer patients. This study aimed to evaluate the impact of anticancer therapy on osteoporosis in Korean breast cancer survivors using data from the National Health Insurance Service-National Health Information Database (NHIS-NHID).

## 2. Materials and Methods

### 2.1. Data Source and Study Population

**Figure 1:**
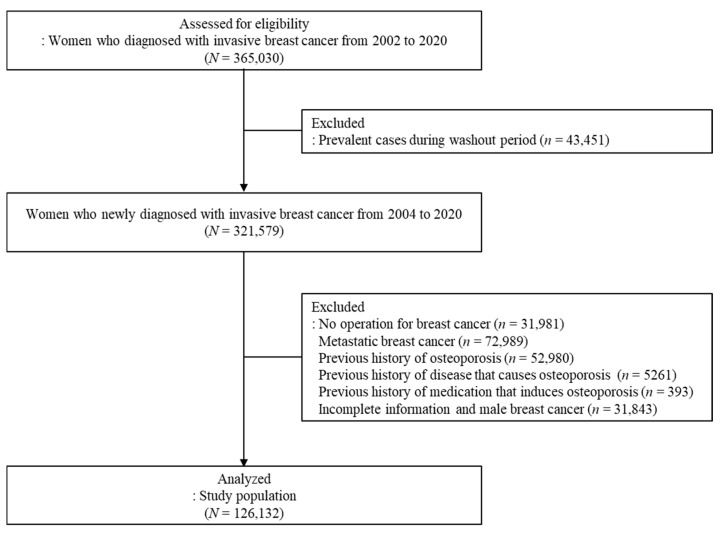
Diagram of the study population.

This study conducted a retrospective cohort analysis using the National Health Insurance Service-National Health Insurance Database (NHIS-NHID) of South Korea, covering the period from 2010 to 2022. The dataset comprised information on qualifications, treatment, medical check-ups, and clinical tables. The database integrates data from a variety of sources, including healthcare providers, hospitals, clinics, and pharmacies, in addition to information derived from the insurance reimbursement process, wherein healthcare providers submit claims for rendered services. The database includes de-identified individual-level demographic information such as age, sex, income level, and medical history and encompasses diagnoses, prescriptions, and procedures documented through inpatient, outpatient, and emergency department visits. The study period was from January 2002 to December 2020. Patients newly diagnosed with breast cancer were defined by combining C50 (code for invasive breast cancer) with V193 (specific claim code issued by the NHIS of Korea for patients with malignant neoplasm). A washout period of 2 years was used to exclude previously diagnosed malignancies. The exclusion criteria included patients without breast cancer surgery, metastatic disease, history of osteoporosis or disease that causes osteoporosis, medication history of drugs that induce osteoporosis (>3 months), and male breast cancer. During the study period, 365,030 patients received the C50 and V193 codes. After applying the exclusion criteria, 126,132 women were analyzed (Figure 1). We obtained the participants’ sex, age, type of insurance, Charlson Comorbidity Index (CCI) based on the International Classification of Diseases, 10th edition (ICD-10) codes (Table S1), treatment, and prescription details. The screening procedures used in this study drew inspiration from those employed in preceding studies [27,28]; nevertheless, there are discernible discrepancies in cohort composition, treatment grouping, and the methodology employed for analysis. Patients who commenced each treatment within 1 year of breast cancer diagnosis and received it for at least 6 months were considered treatment groups. When switching endocrine medications, patients were assigned to the medication group according to the longer or the recently prescribed therapy if the treatment duration was the same. The Institutional Review Board of Kyung Hee University Hospital (KHUH 2021-10-028) approved this study.

### 2.2. Operational Definitions

The operational definition of osteoporosis included (a) prescription of medications exclusively for osteoporosis treatment (Table S2); (b) ICD code for osteoporosis (M80–M82) and prescription of medications related to osteoporosis (additionally indicated for other indications; Table S2); (c) ≥1 hospitalization (≥2 days) or ≥2 outpatient visits (including 1-day hospitalization) with an ICD code for osteoporosis plus older patients (≥65 years old); (d) ≥1 hospitalizations (≥2 days), or ≥2 outpatient visits (including 1-day hospitalization) with an ICD code for osteoporosis plus history of prescribed medications that cause osteoporosis for >3 months (systemic steroid, psychotropic, anticonvulsant, and anticoagulant drugs); (e) ≥1 hospitalizations (≥2 days) or ≥2 outpatient visits (including 1 day hospitalization) with an ICD code for osteoporosis plus history of a disease that causes osteoporosis (Table S3); and (f) osteoporosis-related fractures (Table S4) [29,30,31]. Participants who met at least one of these criteria were defined as having osteoporosis.

Newly-developed osteoporosis was defined as that which occurred >1 year after a breast cancer diagnosis. The following cases were excluded from newly developed osteoporosis: (a) patients with a history of prescribed medications causing osteoporosis for >3 months within 1 year before the diagnosis of osteoporosis, and (b) patients with >2 outpatient visits with a disease that causes osteoporosis within 1 year before the diagnosis of osteoporosis.

### 2.3. Statistical Analysis

The person-years for each patient were calculated from the time of breast cancer diagnosis to the date of osteoporosis occurrence, censoring due to death, loss to follow-up, or last follow-up for osteoporosis. The incidence of osteoporosis was calculated as the number of cases per 1000 person-years. The Cox proportional hazards model was used to evaluate the association between treatment and osteoporosis development, with adjustments for age at diagnosis, type of insurance, and CCI. This model was selected to assess the relative risk of an event occurring over time. A landmark analysis was conducted at 1.5 years to avoid guarantee-time bias, as it takes almost 1.5 years to complete chemotherapy, anti-HER2 therapy, and 6 months of endocrine therapy. Additionally, a sensitivity analysis was performed on multiple landmark time points (1- and 2-year). Treatment groups were defined based on time-dependent variables that occurred before the landmark, and osteoporosis was considered only if it occurred after the landmark. Hazard ratios (HRs) and 95% confidence intervals (CIs) were calculated for each explanatory variable. Survival curves were obtained using the Kaplan–Meier method, and differences between treatments were assessed using the log-rank test. Furthermore, subgroup analysis was performed based on age at breast cancer diagnosis.

Stabilized inverse probability of treatment weighting (IPW) was performed to control confounding bias [32]. Propensity scores were calculated using a multinomial logistic regression model based on age at breast cancer diagnosis, type of insurance, and CCI. It was assumed that the propensity score model was correctly specified and that all relevant confounders were included in the model. Confounding variables were assumed to be effectively controlled using IPW, and treatment allocation was assumed to be fully explained by the adjusted variables.

All statistical analyses were performed using SAS 9.4 software (Statistical Analysis System version 9.4; SAS Institute, Cary, NC, USA), and a *p* < 0.05 (2-tailed test) was considered statistically significant. The ggplot2 package in R version 4.3.0 (www.r-project.org accessed on 22 January 2025) was used for this study [33].

## 3. Results

### 3.1. Participants’ Baseline Characteristics

Breast cancer survivors aged 40–49 years accounted for the highest proportion (40.78%), followed by those aged 50–59 years (34.42%). NHIS subscribers constituted 97% of the patients, and medical aid beneficiaries constituted 2.96%. Patients with CCI scores ≤ 2 accounted for approximately 65%. Approximately 60% of breast cancer survivors received chemotherapy, and 69.26% received endocrine therapy. Approximately 12.6% of breast cancer survivors received anti-HER2 therapy (Table 1). During a median follow-up of 4.22 years, 28,603 osteoporosis cases occurred. The incidence of osteoporosis was 44 cases per 1000 person-years (95% CI, 43.49–44.51).

### 3.2. Effect of Breast Cancer Treatments on Osteoporosis

At the 1.5-year landmark, breast cancer treatments were associated with osteoporosis development adjusted for age at breast cancer diagnosis, type of insurance, and CCI (Table 2). The use of TMX, AIs, chemotherapy, and combinations of these therapies were associated with osteoporosis development. Women treated with AIs had a higher risk of osteoporosis development than those treated with TMX or chemotherapy (HR, 2.53; 95% CI 2.37–2.7, HR, 1.14; 95% CI 1.08–1.21 and HR, 1.3; 95% CI, 1.23–1.38, respectively). Women treated with chemotherapy and anti-HER2 therapy had a higher risk of developing osteoporosis than women treated with chemotherapy alone (HR, 1.41; 95% CI, 1.31–1.52 vs. HR, 1.3; 95% CI, 1.23–1.38, respectively). Women who received AIs following a combination of chemotherapy and anti-HER2 therapy exhibited the highest risk of developing osteoporosis, followed by those receiving AIs (HR, 2.63; 95% CI, 2.41–2.86 and HR, 2.53; 95% CI, 2.37–2.7, respectively). The effect of breast cancer treatment on osteoporosis demonstrated comparable trends across the 1-year, 2-year, and 1.5-year landmark analyses (Table S5).

Subgroup analysis was conducted to examine the effect of treatment on osteoporosis development at the 1.5-year landmark based on the age at breast cancer diagnosis (Table 3). In the group with an age at breast cancer diagnosis of <50 years, all types of treatment, including TMX, AIs, chemotherapy, and combinations of these therapies, significantly elevated the risk of developing osteoporosis. The mean interval between breast cancer diagnosis and subsequent development of osteoporosis was 5.00 ± 3.02 years. The use of AIs was found to be a significant contributing factor to the development of osteoporosis (HR, 4.12; 95% CI, 3.72–4.56). The use of AIs following chemotherapy with or without anti-HER2 therapy was found to be associated with a threefold or greater increased risk of developing osteoporosis in comparison to the no-treatment group. Moreover, the use of TMX and chemotherapy as a monotherapy was associated with an elevated risk of developing osteoporosis (HR, 1.32; 95% CI, 1.20–1.45 and HR, 1.49; 95% CI, 1.35–1.64, respectively).

In the group with an age at breast cancer diagnosis of 50–59 years, treatments except TMX monotherapy were associated with an increased risk of osteoporosis, and the average interval between a diagnosis of breast cancer and the subsequent development of osteoporosis was 4.51 ± 2.72 years. The use of AIs following a combination of chemotherapy and anti-HER2 therapy was found to be associated with the highest risk of osteoporosis (HR, 2.35; 95% CI, 2.05–2.70). The use of TMX as a monotherapy tended to increase the risk of developing osteoporosis; however, it did not reach statistical significance (HR, 1.04; 95% CI, 0.95–1.13).

In the group with an age at breast cancer diagnosis of ≥60 years, the uses of AIs as a monotherapy and AIs following chemotherapy with or without anti-HER2 therapy were associated with the development of osteoporosis. The mean interval between a diagnosis of breast cancer and the subsequent development of osteoporosis was 3.89 ± 2.33 years. The use of TMX as a monotherapy tended to reduce the risk of developing osteoporosis; however, it did not reach statistical significance (HR, 0.93; 95% CI, 0.83–1.04). The use of chemotherapy alone was not found to be associated with the development of osteoporosis (HR, 1.01; 95% CI, 0.89–1.14). The younger the age at which breast cancer is diagnosed, the greater the impact of breast cancer treatment on the subsequent development of osteoporosis. The effect of breast cancer treatments on osteoporosis by age at breast cancer diagnosis exhibited comparable trends across the 1-year, 2-year, and 1.5-year landmark analyses (Table S6).

As anti-HER2 treatment as a monotherapy is not covered by health insurance in Korea, we evaluated its effectiveness by comparing the incidence of osteoporosis in the patients receiving anti-HER2 therapy with those not receiving anti-HER2 therapy, irrespective of whether they received any other treatment. At the 1.5-year landmark analysis, patients treated with anti-HER2 therapy exhibited a heightened risk of developing osteoporosis relative to those treated without anti-HER2 therapy in the groups with age at breast cancer diagnosis of <50 and 50–59 years (HR, 1.23; 95% CI, 1.15–1.31 and HR, 1.22; 95% CI, 1.15–1.29, respectively). However, the use of anti-HER2 therapy did not increase the risk of osteoporosis development in the group with an age at breast cancer diagnosis of ≥60 years (HR, 1.04; 95% CI, 0.96–1.14). The impact of anti-HER2 therapy on osteoporosis by age at breast cancer diagnosis exhibited comparable trends across the 1-year, 2-year, and 1.5-year landmark analyses (Table S7).

**Figure 2:**
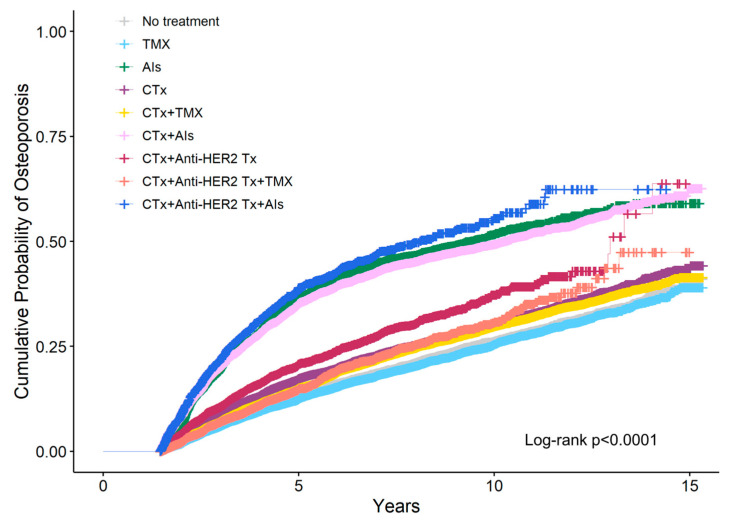
Cumulative probability of osteoporosis according to breast cancer treatment was estimated using inverse probability weighting (IPW) Kaplan–Meier analysis at the 1.5-year landmark. CTx, chemotherapy; TMX, Tamoxifen; AIs, Aromatase Inhibitors; Anti-HER2 Tx, anti-HER2 therapy.

Figure 2 shows the cumulative probability of osteoporosis according to breast cancer treatment estimated using IPW Kaplan–Meier analysis at the 1.5-year landmark. The 5-year cumulative probability was highest for patients who received AIs following a combination of chemotherapy and anti-HER2 therapy (34.8%), followed by AIs monotherapy (34.1%), and AIs following chemotherapy (29.6%).

**Figure 3:**
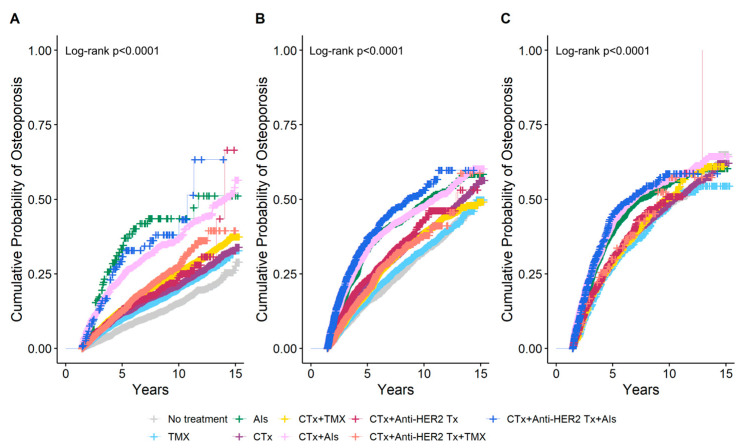
Cumulative probability of osteoporosis according to breast cancer treatment estimated using inverse probability weighting (IPW) Kaplan–Meier analysis at the 1.5-year landmark by age at breast cancer diagnosis. (**A**) Age < 50 years, (**B**) Age 50–59 years, (**C**) Age ≥ 60 years. CTx, chemotherapy; TMX, Tamoxifen; AIs, Aromatase Inhibitors; Anti-HER2 Tx, anti-HER2 therapy.

The cumulative probability of osteoporosis according to breast cancer treatment by age at breast cancer diagnosis was estimated using IPW Kaplan–Meier analysis at the 1.5-year landmark (Figure 3). In the group with an age at breast cancer diagnosis of <50 years, it was observed that the 5-year cumulative probability of osteoporosis was higher in women who received AIs monotherapy at 34.7% than in those who received AIs following a combination of chemotherapy and anti-HER2 therapy at 31.8%. In the group with age at breast cancer diagnosis of ≥60 years, the highest 5-year cumulative probability of osteoporosis was observed in women who received AIs following a combination of chemotherapy and anti-HER2 therapy at 45.1%, followed by those who received AIs after chemotherapy at 42.4%. Among monotherapies, women who received AIs exhibited the highest 5-year cumulative probability of osteoporosis at 39.6%. A comparable trend was observed in the group with an age at breast cancer diagnosis of 50–59 years. The 5-year cumulative probability of osteoporosis with TMX monotherapy differed depending on the age at which breast cancer was diagnosed. In the group with an age at breast cancer diagnosis of <50 years, the 5-year cumulative probability of osteoporosis with TMX monotherapy was 9.9%, which was higher than the probability of 7% observed in women who did not receive any treatment. Conversely, in the group with an age at breast cancer diagnosis of ≥60 years, the 5-year cumulative probability of osteoporosis with TMX monotherapy was 27.5%, which was lower than the probability of 30.7% observed in untreated women.

## 4. Discussion

Our findings indicate that TMX, AIs, chemotherapy, and anti-HER2 therapy are associated with an increased risk of osteoporosis. It is challenging to ascertain the precise reason behind the varying degrees of bone loss induced by different drugs. Nevertheless, estrogen depletion is a major cause of treatment-induced bone loss. Estrogen binds to osteoblast receptors, stimulates osteoprotegerin (OPG) production, and inhibits receptor activators of nuclear factor kappa B ligand (RANKL) [34]. RANKL regulates osteoclast differentiation, activation, and apoptosis. OPG is a decoy receptor for RANKL, thereby suppressing osteoclastogenesis [35,36].

The use of TMX and AIs has been demonstrated to exert an effect on bone health. In a 10-year analysis of the Arimidex, Tamoxifen, Alone or in Combination trial, postmenopausal patients receiving anastrozole had more fractures than those receiving TMX (451 vs. 351; odds ratio 1.33, 95% CI: 1.15–1.55; *p* < 0.0001) [37]. Additionally, anastrozole caused greater BMD decreases in the lumbar spine and total hip (−6.08% and −7.24%, respectively) than TMX (+2.77% and +0.74%, respectively) [38]. Previous studies have demonstrated that AIs decrease BMD in trabecular-rich bone sites by 1–3% annually. This reduction in BMD has been associated with a 3-fold increase in the risk of fracture compared to postmenopausal women without cancer [3,39,40,41,42]. The present study demonstrated that AIs were associated with the development of osteoporosis across all age groups, with a greater impact on osteoporosis than that observed with Tamoxifen or chemotherapy as monotherapy. Notably, the impact of AIs was more pronounced in younger women with early menopause compared to older patients. This is thought to be due to the fact that the rate of bone loss is significantly higher in recently postmenopausal women than in those who have been postmenopausal for a longer period of time. During the menopausal transition period, the average reduction in BMD is approximately 10%. It is estimated that approximately half of women are losing bone at a faster rate, potentially reaching a reduction of 10–20% in the 5–6 years surrounding menopause [43]. In particular, it has been demonstrated that the use of AIs in women who have prematurely menopause as a result of chemotherapy experience had significantly accelerated bone loss when treated with AIs [44]. Our findings indicated that AIs following chemotherapy had a notable impact on the development of osteoporosis, with a more pronounced effect observed in women diagnosed with breast cancer at an earlier age.

TMX is an antagonist in breast cancer cells and an agonist in bone metabolism. The use of TMX in postmenopausal patients with low endogenous estrogen mitigates bone resorption and modestly preserves bone mass loss [19,20,21]. Lumbar spine BMD was reported to decrease by −6.8% in patients with amenorrhea treated with TMX and by −9.5% in those without it [45]. In a 2-year randomized controlled trial, goserelin led to significant BMD loss (mean change, −5%; *p* < 0.001), and TMX reduced GnRH-induced bone loss (mean change, −1.4%; *p* = 0.02) [46]. In this study, the use of TMX tended to reduce the risk of osteoporosis in women who were diagnosed with breast cancer at age ≥ 60 years.

The impact of TMX on osteoporosis may be influenced by menopausal status. TMX progressively decreases BMD in premenopausal patients with a 1.44% mean annual loss in lumbar BMD over a 3-year follow-up period [21]. Ulla et al. demonstrated that the decrease in bone density in the premenopausal TMX group was 2.58 times greater than that in the general population [47]. In the presence of premenopausal estrogen, TMX has estrogenic effects on the hypothalamus-pituitary axis by decreasing follicle-stimulating hormone levels; therefore, it may act as an estrogen antagonist that accelerates bone loss [2,45,48]. The American Cancer Society/American Society of Clinical Oncology guidelines recommend repeating dual-energy X-ray absorptiometry scans every 2 years for premenopausal women receiving TMX and/or GnRH agonists [4]. Nonetheless, the adverse effects of TMX on bone health in premenopausal women are often underestimated in clinical practice. In our study, TMX significantly increased the risk of osteoporosis in patients aged <50 years at breast cancer diagnosis. Inconsistent with our study, a study using Korean Health Insurance and Review Assessment data from 2007 to 2017 found that adjuvant TMX did not increase the risk of osteoporosis in young breast cancer survivors [28]. The authors did not consider switching between different endocrine therapies and time-varying variables. Tzeng et al. showed that TMX reduced the risk of osteoporosis-related fractures by 0.54 times in premenopausal women aged <50 years who received TMX compared to those who did not [49].

Since the publication of the results of the Suppression of Ovarian Function Trial (SOFT) in 2015, adjuvant ovarian function suppression (OFS) has been recommended for premenopausal breast cancer patients with a high risk of recurrence [50]. However, the combined administration of AIs and OFS reduces estrogen levels significantly and promotes bone loss in premenopausal patients [51]. In the Tamoxifen and Exemestane Trial and SOFT, osteoporosis occurred in 13.2% of the patients administered AIs plus OFS and 6.4% of those administered TMX plus OFS [52]. The Austrian Breast and Colorectal Cancer Study Group-12 trial showed that anastrozole plus goserelin caused significantly larger decreases in BMD (−17.3% from baseline at the 36-month follow-up) than TMX plus goserelin (−11.6%; *p* < 0.0001) [53]. Despite the increased frequency of OFS prescriptions, our study found that only approximately 5% of women diagnosed with breast cancer under the age of 60 were treated with OFS. Consequently, the impact of OFS on osteoporosis was not considered in the analysis.

Chemotherapy damages rapidly dividing cells and often causes ovarian dysfunction in premenopausal women, causing premature menopause in 40–95% of patients [54]. Chemotherapy-induced hypogonadism increases bone loss in premenopausal patients with breast cancer, leading to 9.5% and 4.6% rapid bone loss in the lumbar spine and femoral neck, respectively, within 2 years [55,56,57]. In postmenopausal patients, whether chemotherapy is an independent factor for bone loss has not been extensively studied; however, chemotherapy has a deleterious effect on BMD by directly damaging osteoblastic precursors and increasing the activity of osteoclasts, regardless of any effect on ovarian function [55,58,59,60]. Postmenopausal patients who received chemotherapy were likely to have lower BMD than those who did not [59,60]. In our study, a 1.5-year landmark analysis using IPW revealed that chemotherapy increased the risk of osteoporosis by 1.49 times in the group with age at breast cancer diagnosis of <50 years, 1.31 times in those with age at breast cancer diagnosis of 50–59 years, and 1.01 times in those with age at breast cancer diagnosis of ≥60 years, indicating that the effect of chemotherapy on bone loss was more significant in younger women than in older women.

The impact of anti-HER2 treatment on bone health is uncertain. Sanz-Moreno et al. found that anti-HER2 treatment and acquired resistance to anti-HER2 treatment elevated RANK (Receptor activator of nuclear factor kappa) expression levels in HER2-positive cell lines and patients with HER2-positive breast cancer. Additionally, they identified physical and functional interactions between RANK and HER2 [61]. RANK and RANKL (Receptor activator of nuclear factor kappa ligand) play a crucial role in the development and activation of osteoclasts, which subsequently results in bone loss [62]. Our results indicate that women who received anti-HER2 treatment exhibited an elevated risk of developing osteoporosis. Future research is required to investigate the impact of anti-HER2 treatment on bone health to prevent treatment-induced bone loss in breast cancer survivors.

In South Korea, the age group with the highest incidence of newly diagnosed female breast cancer is 40–59 years old [1]. Premenopausal women are not typically at high risk for osteoporosis. It is generally accepted that age-related declines in estrogen levels result in an increased susceptibility to developing primary osteoporosis 10 to 15 years after menopause. However, breast cancer treatment can increase the risk of developing osteoporosis [56]. A previous study on the age distribution of osteoporosis-related fractures in Asian patients with breast cancer showed that such fractures occurred more frequently in patients aged <50 years than in the general population [63]. Some experts support treating premenopausal patients with antiresorptive therapy if they have undergone ovarian suppression and are receiving an AI with a T-score < −1.0 or with a prevalent vertebral fracture [64]. In our study, the majority of breast cancer treatments, including chemotherapy, anti-HER2 therapy, AIs, and TMX, were identified as risk factors for osteoporosis in younger women diagnosed with breast cancer before the age of 50. The mean interval between a diagnosis of breast cancer and the subsequent development of osteoporosis was approximately 5 years. Therefore, attention should be directed toward preventing osteoporosis in young Korean breast cancer survivors.

The findings of this study indicate a necessity to predict the risk of developing osteoporosis in patients receiving breast cancer treatments, irrespective of age. Implementation of preventive strategies is recommended, including screening patients at high risk and performing regular assessments of BMD, as well as lifestyle changes, exercise, calcium and vitamin D intake, and medications. Mitigation of the socioeconomic costs of osteoporosis is achievable through the implementation of these strategies.

This study had some limitations. Firstly, laboratory data, including BMD and body mass index, and lifestyle factors, including smoking and diet, were lacking due to the limitations of data from the NHIS-NHID. Secondly, the effect of chemotherapy on osteoporosis varies depending on the type of agent used; however, we did not investigate drug regimens. Thirdly, it is also important to note that a proportion of the women in the study may have been receiving OFS. However, as previously stated, we did not investigate the potential impact of OFS on the development of osteoporosis due to the relatively low prevalence of this therapy. Thirdly, while we adjusted for confounding variables using Inverse Probability Weighting (IPW), the potential for unmeasured confounders remains, including factors such as menopausal status, body mass index, and family history of osteoporosis [5,60]. These unmeasured confounders were not accounted for in the study, which may introduce potential bias. Lastly, since anti-HER2 monotherapy is not covered by the Korean health insurance system, we evaluated the impact of anti-HER2 therapy on bone health by comparing the occurrence of osteoporosis in patients who were treated with and without anti-HER2 therapy. Further studies are required to more accurately assess the risk of osteoporosis in Korean breast cancer survivors. Notwithstanding these limitations, this study is noteworthy for observing the largest number of patients over a lengthy period among those investigating the association between breast cancer therapy and osteoporosis in Korean patients.

## 5. Conclusions

Breast cancer treatment can have a detrimental impact on bone health, irrespective of the age at breast cancer diagnosis. In younger women, in particular, all breast cancer treatment, including Tamoxifen, contributed to the development of osteoporosis. It is therefore recommended that clinicians inform both older and younger breast cancer patients about the potential for bone loss before commencing anticancer treatment. Furthermore, efforts should be made to prevent the development of osteoporosis.

## Figures and Tables

**Table 1:** Characteristics of the study population.

	Total (*N* = 126,132)	Event (*n* = 28,603)	Incidence Rate (95% CI)
Age at diagnosis			
<30	1081 (0.86)	74 (0.26)	11.72 (9.20–14.72)
30–39	12,809 (10.16)	1235 (4.32)	16.28 (15.38–17.21)
40–49	51,438 (40.78)	8769 (30.66)	31.31 (30.66–31.97)
50–59	43,419 (34.42)	12,267 (42.89)	56.46 (55.46–57.47)
60–69	14,034 (11.13)	5140 (17.97)	90.20 (87.75–92.70)
70–79	2896 (2.30)	1013 (3.54)	85.34 (80.16–90.76)
80–	455 (0.36)	105 (0.37)	59.94 (49.02–72.56)
Type of Insurance			
National Health Insurance	122,350 (97.00)	27,432 (95.91)	43.59 (43.08–44.11)
Medical Aid	3729 (2.96)	1158 (4.05)	56.57 (53.36–59.92)
Others (Unknown)	53 (0.04)	13 (0.05)	40.68 (21.66–69.57)
Charlson Comorbidity Index			
0	17,466 (13.85)	4183 (14.62)	35.75 (34.67–36.85)
1	31,904 (25.29)	7047 (24.64)	38.83 (37.93–39.75)
2	32,644 (25.88)	7212 (25.21)	44.49 (43.47–45.53)
3	21,080 (16.71)	4746 (16.59)	50.00 (48.59–51.44)
4	10,818 (8.58)	2533 (8.86)	55.91 (53.75–58.13)
5+	12,220 (9.69)	2882 (10.08)	58.46 (56.35–60.64)
Treatment			
No treatment	11,403 (9.04)	1848 (6.44)	33.66 (32.14–35.24)
TMX	29,050 (23.03)	4640 (16.22)	30.37 (29.51–31.26)
AIs	9248 (7.33)	2989 (10.45)	87.10 (84.01–90.28)
CTx	20,400 (16.17)	4372 (15.29)	41.14 (39.93–42.37)
CTx+TMX	28,912 (22.92)	6556 (22.92)	37.12 (36.23–38.03)
CTx+AIs	11,203 (8.88)	4583 (16.02)	86.23 (83.75–88.76)
CTx+Anti-HER2 Tx	6961 (5.52)	1547 (22.22)	50.33 (47.86–52.90)
CTx+Anti-HER2 Tx+TMX	6239 (4.95)	1105 (3.86)	35.60 (33.53–37.76)
CTx+Anti-HER2 Tx+AIs	2716 (2.15)	968 (3.38)	92.53 (86.79–98.55)

Values are given as numbers (percent) as categorical variables. CTx, chemotherapy; TMX, Tamoxifen; AIs, Aromatase Inhibitors; Anti-HER2 Tx, anti-HER2 therapy.

**Table 2:** Effect of breast cancer treatments on the development of osteoporosis at the 1.5-year landmark.

	Total (*N* = 103,532) n (%)	Event (*n* = 24,022) n (%)	Crude HR (95% CI)	Adjusted HR (95% CI) *	IPW HR (95% CI)
Treatment					
No treatment	8476 (8.19)	1841 (7.66)	Reference	Reference	Reference
TMX	24,009 (23.19)	3959 (16.48)	0.94 (0.89–0.99)	1.06 (1.00–1.12)	1.14 (1.08–1.21)
AIs	6418 (6.2)	2174 (9.05)	2.66 (2.50–2.83)	1.84 (1.73–1.96)	2.53 (2.37–2.70)
CTx	15,419 (14.89)	3618 (15.06)	1.22 (1.15–1.29)	1.27 (1.20–1.34)	1.30 (1.23–1.38)
CTx+TMX	26,358 (25.46)	5775 (24.04)	1.12 (1.07–1.19)	1.35 (1.28–1.42)	1.39 (1.32–1.47)
CTx+AIs	9101 (8.79)	3595 (14.97)	2.49 (2.35–2.63)	1.92 (1.81–2.03)	2.15 (2.03–2.28)
CTx+Anti-HER2 Tx	5802 (5.6)	1254 (5.22)	1.51 (1.40–1.62)	1.39 (1.30–1.50)	1.41 (1.31–1.52)
CTx+Anti-HER2 Tx+TMX	5673 (5.48)	998 (4.15)	1.13 (1.05–1.22)	1.38 (1.28–1.49)	1.42 (1.32–1.53)
CTx+Anti-HER2 Tx+AIs	2276 (2.2)	808 (3.36)	3.01 (2.77–3.28)	2.26 (2.08–2.46)	2.63 (2.41–2.86)

Values are given as numbers (percent) as categorical variables. * Adjusted for age at diagnosis (<30, 30–39, 40–49, 50–59, 60–69, 70–79, >80), type of insurance (National Health insurance, medical aid, Others (Unknown)) and Charlson comorbidity index status (0, 1, 2, 3, 4, 5+) as categorical variables. HR, hazard ratio; CI, confidence intervals; IPW, inverse probability of treatment weighting; CTx, chemotherapy; TMX, Tamoxifen; AIs, Aromatase Inhibitors; Anti-HER2 Tx, anti-HER2 therapy.

**Table 3:** Effect of breast cancer treatments on the development of osteoporosis at the 1.5-year landmark by age at breast cancer diagnosis.

	Total *N* (%)	Event *n* (%)	Time (Years) *	Crude HR (95% CI)	Adjusted HR (95% CI) **	IPW HR (95% CI)
AGE < 50	54,833	8796	5.00 ± 3.02			
Treatment						
No treatment	3839 (7.00)	477 (5.42)		Reference	Reference	Reference
TMX	16,142 (29.44)	2111 (24.00)		1.46 (1.32–1.62)	1.45 (1.31–1.6)	1.32 (1.20–1.45)
AIs	155 (0.28)	45 (0.51)		3.97 (2.92–5.39)	3.84 (2.83–5.22)	4.12 (3.72–4.56)
CTx	7962 (14.52)	1246 (14.17)		1.48 (1.33–1.64)	1.46 (1.31–1.62)	1.49 (1.35–1.64)
CTx+TMX	19,340 (35.27)	3632 (41.29)		1.89 (1.72–2.08)	1.88 (1.71–2.06)	1.75 (1.60–1.91)
CTx+AIs	890 (1.62)	315 (3.58)		3.24 (2.81–3.74)	3.19 (2.76–3.67)	3.03 (2.76–3.33)
CTx+Anti-HER2 Tx	2132 (3.89)	284 (3.23)		1.74 (1.50–2.01)	1.71 (1.47–1.98)	1.62 (1.43–1.84)
CTx+Anti-HER2 Tx+TMX	4240 (7.73)	644 (7.32)		1.98 (1.76–2.23)	1.94 (1.73–2.19)	1.80 (1.60–2.04)
CTx+Anti-HER2 Tx+AIs	133 (0.24)	42 (0.48)		4.61 (3.36–6.32)	4.39 (3.20–6.02)	3.82 (3.35–4.37)
AGE 50–59	35,562	10,293	4.51 ± 2.72			
Treatment						
No treatment	3060 (8.60)	802 (7.79)		Reference	Reference	Reference
TMX	6995 (19.67)	1554 (15.10)		1.04 (0.95–1.13)	1.04 (0.95–1.13)	1.04 (0.95–1.13)
AIs	2957 (8.32)	937 (9.10)		1.95 (1.77–2.14)	1.93 (1.75–2.12)	1.94 (1.75–2.15)
CTx	5296 (14.89)	1594 (15.49)		1.33 (1.22–1.44)	1.32 (1.21–1.44)	1.31 (1.20–1.43)
CTx+TMX	6429 (18.08)	1879 (18.26)		1.23 (1.14–1.34)	1.23 (1.13–1.34)	1.23 (1.13–1.33)
CTx+AIs	5412 (15.22)	2061 (20.02)		1.93 (1.78–2.09)	1.91 (1.76–2.08)	1.92 (1.75–2.10)
CTx+Anti-HER2 Tx	2605 (7.33)	644 (6.26)		1.44 (1.30–1.60)	1.42 (1.28–1.58)	1.44 (1.29–1.61)
CTx+Anti-HER2 Tx+TMX	1326 (3.73)	311 (3.02)		1.24 (1.09–1.42)	1.23 (1.08–1.41)	1.24 (1.10–1.39)
CTx+Anti-HER2 Tx+AIs	1482 (4.17)	511 (4.96)		2.35 (2.10–2.63)	2.32 (2.07–2.59)	2.35 (2.05–2.70)
AGE ≥ 60	13,137	4933	3.89 ± 2.33			
Treatment						
No treatment	1577 (12.00)	562 (11.39)		Reference	Reference	Reference
TMX	872 (6.64)	294 (5.96)		0.94 (0.82–1.09)	0.94 (0.82–1.09)	0.93 (0.83–1.04)
AIs	3306 (25.17)	1192 (24.16)		1.37 (1.24–1.51)	1.37 (1.24–1.51)	1.35 (1.16–1.57)
CTx	2161 (16.45)	778 (15.77)		1.06 (0.95–1.18)	1.06 (0.95–1.18)	1.01 (0.89–1.14)
CTx+TMX	589 (4.48)	264 (5.35)		1.12 (0.97–1.30)	1.11 (0.96–1.29)	1.04 (0.93–1.16)
CTx+AIs	2799 (21.31)	1219 (24.71)		1.48 (1.34–1.63)	1.47 (1.33–1.62)	1.42 (1.24–1.62)
CTx+Anti-HER2 Tx	1065 (8.11)	326 (6.61)		1.10 (0.96–1.26)	1.09 (0.95–1.25)	1.06 (0.90–1.25)
CTx+Anti-HER2 Tx+TMX	107 (0.81)	43 (0.87)		1.16 (0.85–1.58)	1.15 (0.84–1.56)	1.07 (0.92–1.25)
CTx+Anti-HER2 Tx+AIs	661 (5.03)	255 (5.17)		1.60 (1.38–1.86)	1.59 (1.37–1.84)	1.51 (1.22–1.86)

Values are given as numbers (percent) as categorical variables or numbers ± standard deviation as numeric variables. * *p* value for comparisons of age groups: <0.0001. ** Adjusted for age at diagnosis (<30, 30–39, 40–49, 50–59, 60–69, 70–79, >80), type of insurance (National Health insurance, medical aid, Others (Unknown)) and Charlson comorbidity index status (0, 1, 2, 3, 4, 5+) as categorical variables. HR, hazard ratio; CI, confidence intervals; IPW, inverse probability of treatment weighting; CTx, chemotherapy; TMX, Tamoxifen; AIs, Aromatase Inhibitors; Anti-HER2 Tx, anti-HER2 therapy.

## Data Availability

The data presented in this study are available on request from the corresponding author. The data that support the findings of this study are available from the National Health Insurance Service (NHIS) in South Korea. NHIS data is restricted access to protect the confidentiality of the data and is available upon reasonable request and authorization from the National Health Insurance Administration. Information can be available at https://nhiss.nhis.or.kr (accessed on 20 January 2025) with permission from the NHIS.

## Supplementary Materials

The following supporting information can be downloaded at https://www.mdpi.com/article/10.3390/jcm14030732/s1, Table S1: Charlson comorbidity index (CCI); Table S2: Medications for osteoporosis treatment; Table S3: Diseases that cause osteoporosis; Table S4: ICD-10 codes and operational definition for osteoporosis-related fracture; Table S5: Effect of breast cancer treatments on the development of osteoporosis development at 1 and 2-year landmark; Table S6: Effect of breast cancer treatments on the development of osteoporosis development at 1 or 2-year landmark by age at breast cancer diagnosis; Table S7: Effect of anti-HER2 therapy on the development of osteoporosis by age at breast cancer diagnosis after IPW.
