# Supplementary material for: Effects of Anticancer Therapy on Osteoporosis in Breast Cancer Patients: A Nationwide Study Using Data from the National Health Insurance Service-National Health Information Database
Source: J Clin Med. 2025 Jan 23;14(3):732. doi: 10.3390/jcm14030732 (PMC11818878; doi:10.3390/jcm14030732)
Supplement: Supplementary file 1 [file jcm-14-00732-s001.zip › jcm-3428134-Supplementary.pdf]

Table S1. Charlson comorbidity index (CCI)

Disease	ICD-10 codes	CCI score
Myocardial infarction	I21, I22, I252	1
Congestive heart failure	I43, I50, I099, I110, I130, I132, I255, I420, I425, I426, I427, I428, I429	1
Peripheral vascular disease	I70, I71, I731, I738, I739, I771, I790, I792, K551, K558, K559, Z958, Z959	1
Cerebrovascular disease	G45, G46, I60–I69	1
Dementia	F00–F03, F051, G30, G311	1
Chronic pulmonary disease	I278, I279, J40–J47, J60–J67, J684, J701, J703	1
Rheumatologic disease	M05, M06, M315, M32–M34, M351, M353, M360	1
Peptic ulcer disease	K25–K28	1
Mild liver disease	B18, K73, K74, K700, K701–K703, K709, K713, K714, K715, K717, K760, K762–K764, K768, K769, Z944	1
Diabetes without chronic complication	E100, E101, E106, E108–E111, E116, E118–E121, E126, E128–E131, E136, E138–E141, E146, E148, E149	1
Diabetes with chronic complication	E102–E105, E107, E112–E115, E117, E122–E125, E127, E132–E135, E137, E142–E145, E147	2
Hemiplegia or paraplegia	G81, G82, G041, G114, G801, G802, G830–G834, G839	2
Renal disease	I120, I131, N032–N037, N052–N057, N18, N19, N250, Z490, Z491, Z492, Z940, Z992	2
Any malignancy including leukemia and lymphoma	C00–C26, C30–C34, C37–C41, C43, C45–49, C51–C58, C60–C76, C81–C85, C88, C90–C97	2
Moderate or severe liver disease	I850, I859, I864, I982, K704, K711, K721, K729, K765, K766, K767	3
Metastatic solid tumor	C78–C80	6
Acquired immune deficiency syndrome/human immunodeficiency virus	B20–B22, B24	6

Table S2. Medications for osteoporosis treatment

**Medications exclusively for osteoporosis treatment**
Selective Estrogen Receptor Modifier (19)
Bisphosphonate
Bisphosphonate plus vitamin D combination
Calcitonin
Parathyroid hormone
RANKL (Receptor activator of nuclear factors κB)
Anti-sclerostin antibody
**Additionally indicated for other indications**
Tibolone
Calcium
Calcium plus vitamin D
Vitamin D

Table S3. Diseases that cause osteoporosis

Disease	ICD-10 codes
hypoprolactinemia	E221
panhypopituitarism	E230
premature ovarian failure	E283
Turner syndrome	Q96
Klinefelter syndrome	Q980, Q981, Q982, Q984
Cushing's syndrome	E24
hyperparathyroidism	E21
hyperthyroidism	E05
rheumatoid arthritis	M05, M06, M08, M1200–M1209
End-stage renal disease, ESRD	N18, N19
chronic obstructive pulmonary disease, COPD	J41–J44
inflammatory bowel disease	K50, K51
multiple myeloma	C900
idiopathic hypercalciuria	E835

Table S4. ICD-10 codes and operational definition for osteoporosis-related fracture

		**Osteoporosis-related fracture**
Any site	M80	osteoporosis with pathologic fracture
Spine	M48.4	fatigue fracture of vertebra)
	M48.5	collapsed vertebra, NEC
	S22.0	fracture of thoracic vertebra
	S22.1	multiple fracture of thoracic spine
	S32.0	fracture of lumbar vertebra
Humerus	S42.2	fracture of upper end of humerus
	S42.3	fracture of shaft of humerus
Distal radius	S52.5	fracture of lower end of radius
	S52.6	fracture of lower end of both ulna and radius
Hip	S72.0	fracture of neck of femur
	S72.1	pertrochanteric fracture
Operational definition		Patients who met at least one of these criteria were defined as having an osteoporosis-related fracture<br>age >50 years<br>prescription of medications exclusively for osteoporosis treatment within 1 year before and after the fracture<br>ICD-10 code for osteoporosis and prescription of medications related to osteoporosis within 1 year before and after the fracture<br>history of prescribed medications that cause osteoporosis for >3 months within 1 year before the fracture<br>>2 outpatient visits with a history of a disease that causes osteoporosis within 1 year before the fracture

Table S5. Effect of breast cancer treatments on the development of osteoporosis development at 1 and 2-year landmark

	Landmark 1-year					Landmark 2-year				
	**Total (*N*=114,091) n (%)**	**Event (*n*=28,603) n (%)**	**Crude HR (95% CI)**	**Adjusted HR (95% CI) ***	**After IPW (95% CI)**	**Total (*N*=94,076) n (%)**	**Event (*n*=20,583) n (%)**	**Crude HR (95% CI)**	**Adjusted HR (95% CI) ***	**After IPW (95% CI)**
No treatment	9307 (8.16)	2135 (7.46)	Reference	Reference	Reference	7742 (8.23)	1607 (7.81)	Reference	Reference	Reference
TMX	26,049 (22.83)	4531 (15.84)	0.93 (0.88–0.98)	1.07 (1.01–1.12)	1.15 (1.09-1.21)	22,063 (23.45)	3540 (17.2)	0.96 (0.90–1.01)	1.08 (1.01–1.14)	1.15 (1.09-1.22)
AIs	7697 (6.75)	2880 (10.07)	2.87 (2.71–3.04)	1.99 (1.88–2.11)	2.83 (2.68-3.00)	5537 (5.89)	1815 (8.82)	2.68 (2.50–2.86)	1.84 (1.72–1.98)	2.57 (2.39-2.75)
CTx	17,069 (14.96)	4338 (15.17)	1.27 (1.21–1.34)	1.33 (1.26–1.40)	1.37 (1.30-1.44)	13,997 (14.88)	3090 (15.01)	1.17 (1.11–1.25)	1.22 (1.15–1.30)	1.26 (1.19-1.34)
CTx+TMX	28,120 (24.65)	6543 (22.88)	1.12 (1.06–1.17)	1.35 (1.29–1.42)	1.40 (1.33-1.47)	24,608 (26.16)	5158 (25.06)	1.14 (1.08–1.20)	1.36 (1.28–1.44)	1.40 (1.32-1.48)
CTx+AIs	10,607 (9.30)	4574 (15.99)	2.67 (2.53–2.81)	2.07 (1.96–2.18)	2.29 (2.17-2.42)	7910 (8.41)	2855 (13.87)	2.30 (2.16–2.45)	1.77 (1.66–1.88)	1.94 (1.82-2.07)
CTx+Anti-HER2 Tx	6531 (5.72)	1534 (5.36)	1.53 (1.44–1.64)	1.42 (1.33–1.52)	1.44 (1.34-1.54)	5139 (5.46)	1024 (4.97)	1.46 (1.35–1.58)	1.35 (1.24–1.46)	1.36 (1.26-1.48)
CTx+Anti-HER2 Tx+TMX	6075 (5.32)	1104 (3.86)	1.06 (0.99–1.14)	1.31 (1.21–1.41)	1.34 (1.25-1.44)	5202 (5.53)	878 (4.27)	1.17 (1.08–1.27)	1.42 (1.31–1.54)	1.45 (1.34-1.57)
CTx+Anti-HER2 Tx+AIs	2636 (2.31)	964 (3.37)	2.86 (2.65–3.08)	2.17 (2.01–2.34)	2.69 (2.49-2.91)	1878 (2.00)	616 (2.99)	2.84 (2.59–3.12)	2.12 (1.93–2.33)	2.49 (2.26-2.74)

Values are given as numbers (percent) as categorical variables. * Adjusted for age at diagnosis (<30, 30-39, 40-49, 50-59, 60-69, 70-79, >80), type of insurance (National Health Insurance, Medical Aid, Others (Unknown)) and Charlson comorbidity index status (0,1,2,3,4,5+) as categorical variables. HR, hazard ratio; CI, confidence intervals; IPW, inverse probability of treatment weighting; CTx, chemotherapy; TMX, Tamoxifen; AIs, Aromatase Inhibitors; Anti-HER2 Tx, anti-HER2 therapy

Table S6. Effect of breast cancer treatments on the development of osteoporosis development at 1 or 2-year landmark by age at breast cancer diagnosis

	Landmark 1-year						Landmark 2-year					
	Total n (%)	Event n (%)	Time (Years) *	Crude HR (95% CI)	Adjusted HR (95% CI) **	After IPW (95% CI)	Total n (%)	Event n (%)	Time (Years) *	Crude HR (95% CI)	Adjusted HR (95% CI) **	After IPW (95% CI)
AGE <50	59,262	10,078	4.51 ± 3.09				50,617	7,753	5.44 ± 2.94			
No treatment	4133 (6.97)	531 (5.27)		Reference	Reference	Reference	3572 (7.06)	424 (5.47)		Reference	Reference	Reference
TMX	17,612 (29.72)	2446 (24.27)		1.51 (1.38–1.66)	1.50 (1.36–1.65)	1.37 (1.26-1.5)	14,799 (29.24)	1884 (24.3)		1.48 (1.33–1.65)	1.47 (1.32–1.63)	1.33 (1.2-1.47)
AIs	174 (0.29)	52 (0.52)		4.30 (3.23–5.71)	4.16 (3.13–5.53)	4.84 (4.4-5.31)	151 (0.3)	43 (0.55)		3.91 (2.86–5.35)	3.79 (2.77–5.2)	4.19 (3.76-4.67)
CTx	8638 (14.58)	1458 (14.47)		1.57 (1.42–1.73)	1.55 (1.40–1.71)	1.59 (1.45-1.74)	7357 (14.53)	1088 (14.03)		1.44 (1.28–1.61)	1.42 (1.27–1.59)	1.45 (1.31-1.61)
CTx+TMX	20,639 (34.83)	4125 (40.93)		1.94 (1.77–2.12)	1.92 (1.75–2.10)	1.8 (1.65-1.96)	18,028 (35.62)	3215 (41.47)		1.88 (1.70–2.08)	1.87 (1.69–2.07)	1.74 (1.58-1.91)
CTx+AIs	1029 (1.74)	367 (3.64)		3.43 (3.00–3.92)	3.38 (2.96–3.86)	3.22 (2.94-3.52)	802 (1.58)	268 (3.46)		3.06 (2.62–3.56)	3.01 (2.58–3.51)	2.69 (2.43-2.98)
CTx+Anti-HER2 Tx	2339 (3.95)	333 (3.30)		1.77 (1.54–2.03)	1.74 (1.51–1.99)	1.66 (1.47-1.86)	1915 (3.78)	231 (2.98)		1.64 (1.40–1.93)	1.62 (1.38–1.90)	1.54 (1.35-1.77)
CTx+Anti-HER2 Tx+TMX	4548 (7.67)	715 (7.09)		1.91 (1.71–2.14)	1.88 (1.67–2.10)	1.75 (1.56-1.97)	3882 (7.67)	568 (7.33)		2.04 (1.79–2.31)	2.00 (1.77–2.27)	1.86 (1.64-2.12)
CTx+Anti-HER2 Tx+AIs	150 (0.25)	51 (0.51)		4.75 (3.57–6.34)	4.54 (3.41–6.06)	4.29 (3.80-4.85)	111 (0.22)	32 (0.41)		4.17 (2.91–5.97)	3.98 (2.78–5.70)	3.68 (3.18-4.25)
AGE 50–59	39,495	12,267	3.98 ± 2.78				35,562	10,293	4.99 ± 2.67			
No treatment	3391 (8.59)	934 (7.61)		Reference	Reference	Reference	3060 (8.60)	802 (7.79)		Reference	Reference	Reference
TMX	7517 (19.03)	1756 (14.31)		1.02 (0.94–1.11)	1.02 (0.94–1.11)	1.02 (0.95-1.11)	6995 (19.67)	1554 (15.10)		1.03 (0.95–1.13)	1.03 (0.94–1.13)	1.03 (0.94-1.13)
AIs	3532 (8.94)	1257 (10.25)		2.16 (1.99–2.35)	2.14 (1.96–2.33)	2.17 (1.98-2.37)	2957 (8.32)	937 (9.10)		1.92 (1.74–2.13)	1.90 (1.72–2.10)	1.91 (1.71-2.13)
CTx	5920 (14.99)	1897 (15.46)		1.37 (1.27–1.49)	1.37 (1.27–1.48)	1.36 (1.26-1.48)	5296 (14.89)	1594 (15.49)		1.24 (1.14–1.36)	1.24 (1.13–1.36)	1.23 (1.12-1.35)
CTx+TMX	6856 (17.36)	2126 (17.33)		1.23 (1.14–1.33)	1.23 (1.13–1.32)	1.22 (1.13-1.32)	6429 (18.08)	1879 (18.26)		1.24 (1.13–1.35)	1.24 (1.13–1.35)	1.23 (1.13-1.34)
CTx+AIs	6200 (15.70)	2556 (20.84)		2.03 (1.89–2.19)	2.02 (1.87–2.18)	2.02 (1.86-2.20)	5412 (15.22)	2061 (20.02)		1.75 (1.6–1.91)	1.74 (1.59–1.9)	1.74 (1.58-1.92)

CTx+Anti-HER2 Tx	2942 (7.45)	787 (6.42)		1.47 (1.33–1.61)	1.45 (1.32–1.59)	1.47 (1.32-1.63)	2605 (7.33)	644 (6.26)		1.39 (1.24–1.55)	1.37 (1.22–1.53)	1.39 (1.23-1.57)
CTx+Anti-HER2 Tx+TMX	1417 (3.59)	344 (2.80)		1.17 (1.03–1.32)	1.16 (1.03–1.31)	1.16 (1.04-1.29)	1326 (3.73)	311 (3.02)		1.26 (1.09–1.44)	1.24 (1.08–1.43)	1.25 (1.10-1.41)
CTx+Anti-HER2 Tx+AIs	1720 (4.35)	610 (4.97)		2.25 (2.03–2.49)	2.22 (2.00–2.46)	2.26 (1.99-2.57)	1482 (4.17)	511 (4.96)		2.14 (1.89–2.42)	2.1 (1.86–2.38)	2.14 (1.83-2.51)
AGE ≥60	15,534	6,258	3.32 ± 2.34				13,137	4,933	4.38 ± 2.31			
No treatment	1783 (11.63)	670 (10.71)		Reference	Reference	Reference	1577 (12)	562 (11.39)		Reference	Reference	Reference
TMX	920 (6)	329 (5.26)		0.93 (0.82–1.06)	0.93 (0.81–1.06)	0.91 (0.82-1.01)	872 (6.64)	294 (5.96)		0.97 (0.83–1.13)	0.97 (0.83–1.13)	0.97 (0.85-1.09)
AIs	3991 (26.03)	1571 (25.1)		1.45 (1.32–1.58)	1.45 (1.32–1.58)	1.43 (1.25-1.63)	3306 (25.17)	1192 (24.16)		1.39 (1.25–1.55)	1.39 (1.25–1.56)	1.37 (1.16-1.62)
CTx	2,511 (16.38)	983 (15.71)		1.14 (1.03–1.25)	1.13 (1.03–1.25)	1.09 (0.97-1.21)	2161 (16.45)	778 (15.77)		1.05 (0.93–1.18)	1.04 (0.93–1.17)	1.00 (0.87-1.14)
CTx+TMX	625 (4.08)	292 (4.67)		1.09 (0.95–1.25)	1.08 (0.95–1.24)	1.03 (0.93-1.13)	589 (4.48)	264 (5.35)		1.17 (0.99–1.37)	1.16 (0.99–1.35)	1.08 (0.95-1.21)
CTx+AIs	3378 (22.03)	1651 (26.38)		1.64 (1.50–1.79)	1.63 (1.49–1.78)	1.58 (1.41-1.78)	2799 (21.31)	1219 (24.71)		1.37 (1.23–1.53)	1.37 (1.22–1.53)	1.31 (1.13-1.51)
CTx+Anti-HER2 Tx	1250 (8.15)	414 (6.62)		1.13 (1.00–1.28)	1.12 (0.99–1.27)	1.1 (0.95-1.27)	1065 (8.11)	326 (6.61)		1.07 (0.92–1.25)	1.07 (0.92–1.24)	1.05 (0.87-1.26)
CTx+Anti-HER2 Tx+TMX	110 (0.72)	45 (0.72)		1.04 (0.77–1.41)	1.03 (0.76–1.40)	0.98 (0.85-1.12)	107 (0.81)	43 (0.87)		1.18 (0.84–1.64)	1.16 (0.83–1.63)	1.09 (0.92-1.29)
CTx+Anti-HER2 Tx+AIs	776 (5)	303 (4.84)		1.50 (1.31–1.72)	1.49 (1.30–1.70)	1.43 (1.18-1.72)	661 (5.03)	255 (5.17)		1.58 (1.34–1.86)	1.56 (1.32–1.84)	1.48 (1.16-1.89)

Values are given as numbers (percent) as categorical variables. * P value for comparisons of age groups: < 0.0001. ** Adjusted for age at diagnosis (<30, 30-39, 40-49, 50-59, 60-69, 70-79, >80), type of insurance (National Health Insurance, Medical Aid, Others (Unknown)) and Charlson Comorbidity Index status (0,1,2,3,4,5+) as categorical variables. HR, hazard ratio; CI, confidence intervals; IPW, inverse probability of treatment weighting; CTx, chemotherapy; TMX, Tamoxifen; AIs, Aromatase Inhibitors; Anti-HER2 Tx, anti-HER2 therapy

Table S7. Effect of anti-HER2 therapy on the development of osteoporosis by age at breast cancer diagnosis after IPW

			Total	AGE <50	AGE 50–59	AGE ≥60
	**Total**	**Event**	**HR (95% CI) ***	**HR (95% CI) ***	**HR (95% CI) ***	**HR (95% CI) ***
Landmark 1-year						
No Anti-HER2 Tx	110,216	25,085	Reference	Reference	Reference	Reference
Anti-HER2 Tx	16,616	3518	1.12 (1.08-1.16)	1.17 (1.10-1.24)	1.16 (1.10-1.23)	0.96 (0.89-1.04)
Landmark 1.5-year						
No Anti-HER2 Tx	90,059	21,013	Reference	Reference	Reference	Reference
Anti-HER2 Tx	13,473	3009	1.18 (1.14-1.23)	1.23 (1.15-1.31)	1.22 (1.15-1.29)	1.04 (0.96-1.14)
Landmark 2-year						
No Anti-HER2 Tx	82,096	18,105	Reference	Reference	Reference	Reference
Anti-HER2 Tx	11,980	2478	1.18 (1.13-1.23)	1.23 (1.15-1.32)	1.2 (1.12-1.28)	1.05 (0.95-1.15)

Values are given as numbers (percent) as categorical variables. * Adjusted for age at diagnosis (<30, 30-39, 40-49, 50-59, 60-69, 70-79, >80), type of insurance (National Health Insurance, Medical Aid, Others (Unknown)) and Charlson Comorbidity Index status (0,1,2,3,4,5+) as categorical variables. HR, hazard ratio; CI, confidence intervals; IPW, inverse probability of treatment weighting; Anti-HER2 Tx, anti-HER2 therapy
